# Treatment-seeking behaviours of malaria patients versus non-malaria febrile patients along China-Myanmar border

**DOI:** 10.1186/s12936-023-04747-4

**Published:** 2023-10-14

**Authors:** Jian-Wei Xu, Dao-Wei Deng, Chun Wei, Xing-Wu Zhou, Jian-Xiong Li

**Affiliations:** Yunnan Institute of Parasitic Diseases, Yunnan Provincial Key Laboratory of Vector-Borne Disease Control and Research; Training Base of International Scientific Exchange and Education in Tropical Diseases for South and Southeast Asia; Yunnan International Joint Laboratory of Tropical Infectious Diseases, Puer, 665000 China

**Keywords:** Malaria, Treatment-seeking behaviour, Laboratory-based diagnosis, China-Myanmar border

## Abstract

**Background:**

Appropriate malaria treatment-seeking behaviour (TSB) is critical for timely detecting malaria, prompt treatment, and prevention of onward transmission of the disease in a community. This study aimed to compare treatment-seeking behaviours between malaria patients and non-malaria febrile patients, and to analyse the factors associated with appropriate TSB along the China-Myanmar border.

**Methods:**

A cross-sectional study was carried out to investigate the appropriate TSB of microscopy-confirmed malaria patients versus non-malaria febrile (NMF) patients. An unconditional logistic regression analysis (LRA) was used to identify factors associated with appropriate TSB.

**Results:**

Among 223 malaria patients and 446 NMF patients, 129 (57.8%) of the malaria patients versus 163 (36.5%) of the NMF patients firstly sought treatment in health facilities without laboratory testing for malaria (*P* < 0.0001). A total of 85(38.1%) of the malaria patients versus 278 (62.3%) of the NMF patients had appropriate TSB, namely, seeking treatment in health facilities with laboratory testing for malaria within 48 h (*P* < 0.0001). Multivariate LRA identified that the malaria patients with Chinese nationality had less appropriate TSB compared to those with other nationalities (adjusted odds ratio [AOR]: 0.21, 95% confidence interval CI 0.07–0.68, *P* = 0.0097), and malaria patients residing in urban areas had more appropriate TSB compared to those living in rural areas (AOR: 2.16, 95%CI 1.06–4.39, P = 0.0337).

**Conclusions:**

TSB was not appropriate in malaria patients. Chinese citizenship and rural residence were two independent factors associated with inappropriate malaria TSB. It is urgently necessary to improve appropriate malaria TSB through effective campaigns of information, education, and communication for malaria control in Myanmar and preventing reestablishment of malaria transmission in Yunnan, China.

**Supplementary Information:**

The online version contains supplementary material available at 10.1186/s12936-023-04747-4.

## Background

Malaria is one of the leading public health problems, and nearly half of the world’s population is still at risk of infection. Malaria control has stagnated since 2015 and worsened during the COVID-19 pandemic [1]. A total of 247 million cases and 619,000 deaths from malaria were reported in 2021 [2]. The cross-border transmission of malaria remains a regional impediment to the goal of the World Health Organization (WHO) malaria elimination in the Great Mekong Subregion (GMS) by 2030 [3, 4]. Myanmar reported significant numbers of cases and deaths in six countries of the GMS. In 2022, Myanmar tested a total of 2,257,126 febrile patients for malaria, and reported a total of 125,704 confirmed malaria cases (22,486* Plasmodium falciparu*m + mixed and 103,218 *Plasmodium vivax*) and 13 deaths of malaria patients [2, 5]. Yunnan (China) is a unique province with the malaria ecology and vector system similar to those of five other countries [6–8]. In the early years of this century, the Yunnan border area was still highly endemic for malaria. In 2003, there were a total of 10,349 malaria cases, and a 17.1 per 10,000 person-years of annual parasite incidence (API) in the border area. Intensive interventions reduced the overall API to 0.6 per 10,000 person-years in the Yunnan border area in 2013. The effective cross-border collaboration dramatically reduced the malaria burden by 90% in the border areas of Myanmar side. From 2014 onward, the comprehensive strategy successfully prevented reintroduction of malaria in Yunnan, and then China was certificated malaria free by the WHO [9, 10].

Clearance of malaria parasite reservoirs with drug-based interventions is the primary strategy for malaria control and elimination [9–11]. Early diagnosis and prompt treatment of malaria cases can improve prognosis for malaria patients and reduce the infectious reservoir, while simultaneously containing the spread of drug resistance. Therefore, the early diagnosis and prompt treatment are believed to be critical for the success and the sustainability of malaria interventions [12, 13]. In the northern Myanmar, an investigation reported that only 32.0% of febrile patients among the Wa people sought treatment within 24 h, and most of them (79.6%) initially used the drug retail sector [12]. Civil wars, political conflicts, ethnic issues, and stagnant social development are regarded as the cause of fragile health systems, and then low health awareness is listed as one of the contributing factors to the poor health status, and the disparity in health-seeking behaviours for fever between rural and urban residents in the northern Myanmar [14, 15]. Most parts of Yunnan are tropical and subtropical regions with high malaria receptivity. In the elimination setting with high malaria receptivity, delays in seeking appropriate malaria treatment will increase the risk of reestablishment of malaria transmission [6]. It is, therefore, important to ensure that people can access appropriate malaria treatment in time.

The China Information System for Disease Control and Prevention (CISDCP) reports data of malaria cases in real time. However, the CISDCP can only report the times of patient attendance at a health facility, not the accurate time of onset of symptoms and treatment-seeking behaviours [16]. Currently, the data on treatment-seeking behaviours (TSB) of febrile patients are still lacking along the China- Myanmar border. To fill this gap and support the regional commitment to achieve zero local transmission by 2030, and prevent reestablishment of malaria transmission in Yunnan, this study compared TSB between microscopy-confirmed malaria patients and non-malaria febrile patients, and then analysed factors associated with appropriate TSB.

## Methods

### Study area

The 2186 km China-Myanmar boundary consists of two sections: 189 km of the Tibet (China)-Myanmar and 1997 km of the Yunnan (China)—Myanmar border. Due to the high altitude and frigid climate, malaria cannot be transmitted across the Tibet-Myanmar border. There are 18 counties under six prefectures in Yunnan along the border between Yunnan and Myanmar. A population of 5.6 million is living in the Yunnan border area, where malaria-free status has been maintained since the last indigenous *P. vivax* malaria case was reported in April 2016 [6]. The risk of malaria reintroduction is also very low in Nujiang Prefecture of the Yunnan due to its cold climate resulting from high altitude and seasonal snowfall on the mountains.

**Fig. 1:**
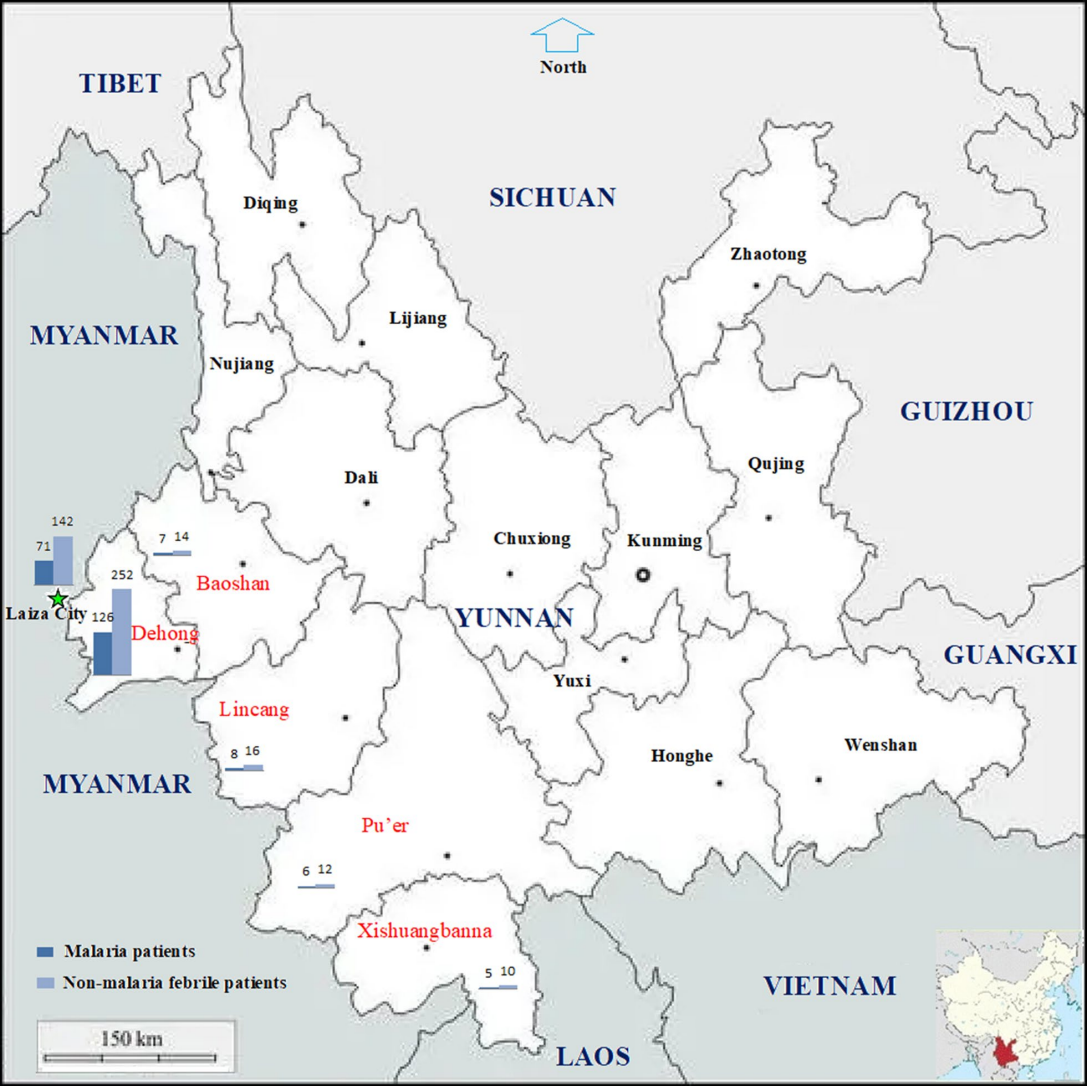
Study site locations, altitudes and number of subjects enrolled at each site

There are 23 townships under Kachin State and Shan State in the Myanmar side of the international border. A total 6321 malaria cases (5894 in Kachin, 286 in Northern Shan, and 141 in Eastern Shan) were reported from these townships with a population of 1.5 million in 2016 [17]. Most parts of the Myanmar border area are managed by the five local ethnic minority governments: Kachin Special Region I (KR1), Kachin Special Region II (KR2), Kokang Autonomous Region (KAR), Shan Special Region II (SR2), and Eastern Shan Special Region IV (SR4). The Laiza City and the surrounding areas in the KR2 are an important malaria hot spot [17]. Based on the epidemiology of the local malaria situation [17–20], this study was conducted in five prefectures in the Yunnan of China: Dehong, Baoshan, Lincang, Pu'er, and Xishuangbanna, as well as in the KR2 of Myanmar. The study sites in the Yunnan were originally hyperendemic areas with high receptivity and a high risk of malaria reintroduction [8]. As one of the enrollment criteria, these participant health facilities must routinely perform microscopy for malaria diagnosis. At last, 37 health facilities in the five prefectures of the Yunnan and the Laiza City Hospital in the KR2 of Myanmar conducted this study (Fig. 1).

### Study design and recruitment

**Fig. 2:**
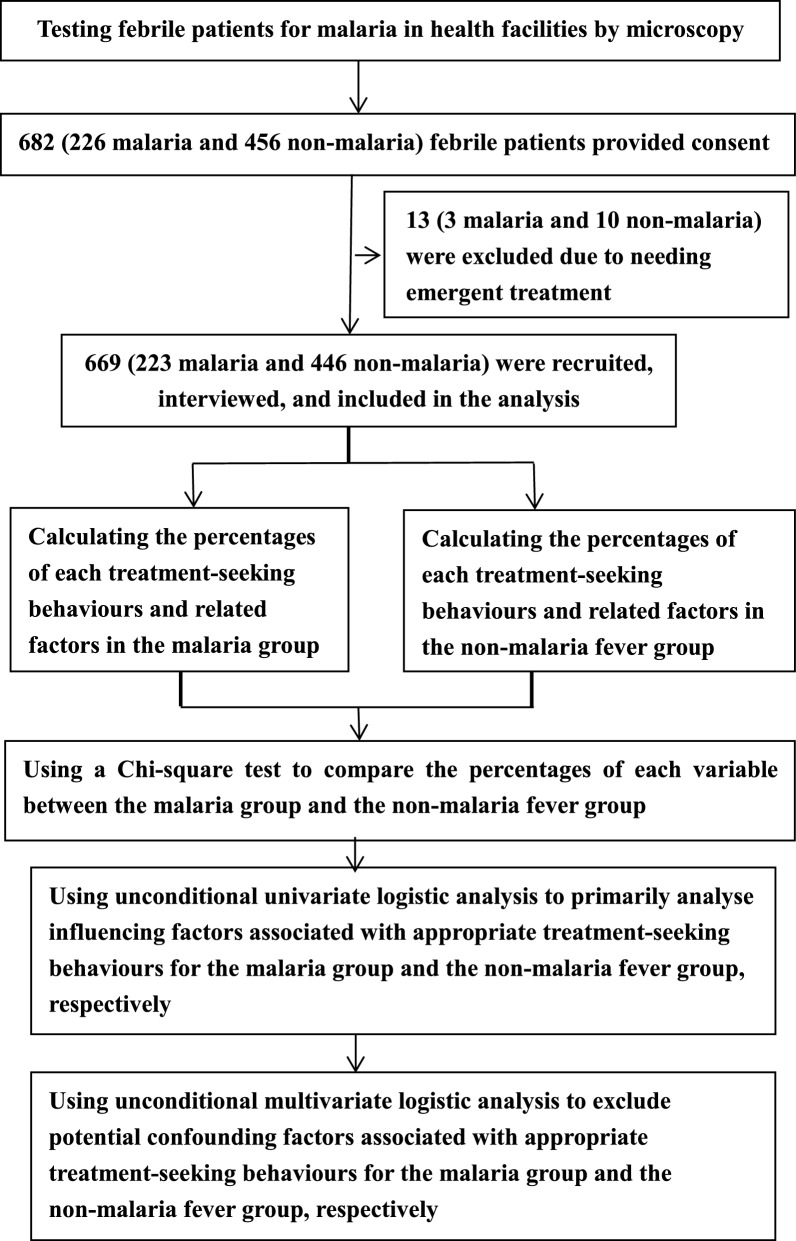
Enrollment of participants and analyses of data

In this cross-sectional study, the microscopy-confirmed malaria cases and the microscopy-excluded non-malaria febrile (NMF) patients who attended the same health facility were recruited (Fig. 2). Following one malaria case enrolled, two NMF patients were recruited within a week. This recruiting approach was repeated till to reaching the required simple size. The sample size was calculated using a 95% two-sided confidence level, 80% power, 20% of malaria cases with exposure to attending a health facility with laboratory testing for malaria within 48 h, and 10% of NMF patients with the same exposure in Epi Info 7.0 (Centers for Disease Control and Prevention, USA). The sample size obtained was at least a total of 158 malaria cases and 316 NMF patients. From May 2016 to October 2017, the research subjects were enrolled, and then an expert microscopist with the WHO Malaria Microscopy Level One Certificate re-read the blood slides to further confirm malaria cases and exclude malaria from the NMF patients.

### Malaria diagnosis and treatment and data collection

Thin and thick smears for patients with documented fever (axillary temperature ≥ 37.5 ℃) or a history of fever in the previous 48 h were prepared and stained with Giemsa in the study health facilities. Laboratory technicians obtained informed consent, and then conducted microscopy for malaria parasites. Based on the results of microscopy, patients were assigned to either the confirmed malaria group or the NMF group. Patients were excluded from this study if they were in severe clinical conditions, as were those without informed consent, who were also excluded (Fig. 2). As a quality control, all blood slides of research subjects were re-read by an expert microscopist with the WHO Malaria Microscopy Capacity Level One Certificate for further confirmation, namely, to exclude subjects with false negativity and false positivity. Anti-malarial treatment is free of charge for all malaria cases, provided by the National Malaria Elimination Programme in China and the Malaria Project of the Global Fund to Fight AIDS, Tuberculosis, and Malaria in Myanmar.

A paper-based questionnaire was pre-tested for validation. Before the study, laboratory technicians who acted as interviewers underwent intensive training to ensure that the interviewers from the Laiza City Hospital, who were fluent in both Kachin and Chinese, could ask each question in Kachin and then fill out the questionnaire in Chinese. The questionnaire was administered to each subject in Mandarin in 37 health facilities in China, and in the Kachin ethnic language at the Laiza City Hospital in Myanmar. For the child participants, their parents or guardians helped to answer the questions. The questionnaire consisted of 36 questions on treatment-seeking behaviour for fever, socio-demographic characteristics, education, main sources of cash, places of residence, housing conditions, items of durable assets, malaria awareness and knowledge, and family decision-making. According to the principal household components, a quintile Family Wealth Index (FWI) was constructed in the questionnaire, namely, most poor, mid-low, middle, mid-high, and least poor (Additional file 1: Table S1) [21].

### Statistical analysis

Data were entered and cleaned in Excel 2007 and then analysed in Epi Info 7.2. First, the proportions of TSB, including the time intervals between the onset of symptoms and attendance of health facilities with laboratory testing for malaria, self-medication, and other sites visited for treatment prior to attending the health facilities with laboratory testing, were calculated for the malaria group and the NMF group, respectively. Second, the proportions of TSB between the malaria group and the NMF group were compared using a two-tailed Fisher exact chi-squared test, with of P < 0.05 were considered significant. The same statistical analysis above was conducted for the data of the subsample collected in China and in Myanmar, respectively. Third, influence factors associated with appropriate TSB were analysed using unconditional multivariate logistic regression analysis to control for confounding factors (Fig. 2).

An ideal TSB for malaria case patients should be to visit a health facility within 24 h [23]. However, the primary analysis results showed that the percentage of malaria case patients seeking treatment in a health facility with laboratory testing within 24 h was too low to be statistically significant. Most of the malaria cases were *P. vivax* along the China-Myanmar border. Taking into consideration of these two factors, the appropriate TSB was defined as seeking treatment in a health facility with laboratory testing (microscopy or rapid diagnostic test [RDT] or both) for malaria within 48 h. Otherwise, the TSB was deemed inappropriate. In logistic regression analysis (LRA), the outcome variable was the appropriate TSB. The independent variables were variables of socio-demographic characteristics, education, main cash sources, residing locations, housing condition, FWI, malaria awareness and knowledge, and family decision. Non-response answers were treated as missing values and, therefore, excluded from the analysis [8].

## Results

### Characteristics of participants

A total of 669 participants, comprising 223 malaria cases and 446 NMF patients, were enrolled in this study. Of them, 489 (73.1%) were male, and the mean age was 30.6 years old (median: 29.0, range: 1–76). Of the 223 malaria cases, 210 (94.2%) were *P. vivax*, 11 (4.9%) were *P. falciparum* and two (0.9%) were mixed infections with *P. vivax and P. falciparum*. The proportions of subjects with Chinese nationality and ≤ 6 years of formal schooling were not significantly different between the malaria group and the NMF group (P > 0.05). The proportions of participant households with FWI ≤ 3 were statistically significant (P = 0.0067) between the two groups of patients. Demographic characteristics and education were similar between the two groups (P > 0.05), but the families of the malaria cases were poorer than those of the NMF patients (Table 4 and Additional file 1: Table S2).

Demographic characteristics of the 456 subjects recruited in China were similar to of the overall sample (Additional file 1: Tables S2 and S3). Based on the CISDCP, all 152 malaria cases that were enrolled in China were imported, including 141 (92.8%) *P. vivax* cases, 10 (6.6%) *P. falciparum* cases, and 1 (0.7%) mixed infection with *P. vivax* and *P. falciparum*. The number of participant households with FWI ≤ 3 was 72 (47.4%) in the malaria group and 79 (26.0%) in the NMF group, showing significance (< 0.0001).The malaria cases received less schooling, and their families were poorer in economic status compared to the NMF patients (Additional file 1: Table S3).

Among 213 participants (71 cases and 142 controls) recruited in Myanmar, 129 (60.6%) were male, and the mean age was 22.0 years old (median: 21.0, range: 2–67). Of the 71 malaria cases, 69 (97.2%) were *P. vivax*, 1 (1.4%) was *P. falciparum,* and 1 was (1.4%) a mixed infection with *P. vivax* and *P. falciparum*. Only one (1.4%) of the malaria cases and 4 (2.8%) of the NMF patients were Chinese citizens (P = 0.8729). The education level and family economic status were similar between the two groups of patients (Additional file 1: Table S4).

### Treatment-seeking behaviours

The proportion of malaria patients who initially used self-medication was bigger than that of the NMF patients (P = 0.0012). Before attending a health facility with laboratory testing for malaria, the proportion of malaria cases who initially sought medication in sites without laboratory-based diagnosis (including drug stores, village health posts, and private clinics) was also higher than that of the NMF patients (P < 0.0001) (Table 1). Only 14.4% of the malaria cases sought treatment within 24 h, compared to 37.2% of the NMF patients (P < 0.0001). The accumulated proportion of those seeking treatment in health facilities with laboratory testing within 48 h was significantly different between the two groups of patients (P < 0.0001). Most malaria cases (88.3%) sought treatment for malaria in health facilities with laboratory testing within a week (Table 1); however, one malaria patient enrolled in China sought treatment after 31 days of symptom onset.

**Table 1:** Treatment-seeking behaviours between malaria cases and non-malaria fever patients

Treatment-seeking behaviours	Malaria cases (%, 95%CI) N = 223	% Accumulated	Non-malaria febrile patients (%, 95%CI) N = 446	% Accumulated	*P value*
Time prior to attending health facilities with laboratory testing (hours)					
≤ 24(1 day)	32 (14.4, 10.0–19.7)	14.4	167 (37.4, 32.8.0–41.9)	37.4	< 0.0001
25–48(2 days)	53 (23.8, 18.3–29.9)	38.1	111 (24.9, 21.2–29.5)	62.3	0.7753
49–72(3 days)	38 (17.0, 12.4–22.6)	55.2	104 (23.3, 19.5–27.6)	85.6	0.0764
73–168(7 days)	74 (33.2, 27.0–39.8)	88.3	59 (13.2, 10.3–16.8)	98.8	< 0.0001
169–360 (15 days)	21 (9.4, 5.9–14.0)	97.8	4 (0.9, 0.3–2.4)	99.8	< 0.0001
> 360 (15 days)	5 (2.2, 0.7–5.2)	100	1 (0.2, 0.01–2.4)	100	0.0296
Self-medication prior to attending health facilities with laboratory testing					
Yes	67 (30.0, 24.1–36.5)	30.0	83 (18.6, 15.2–22.6)	18.6	0.0012
No	156 (70.0, 63.5–75.9)	100	363 (81.4, 77.4–84.8)	100	0.0012
Visited sites without laboratory testing for malaria prior to seeking appropriate treatment					
No	94 (42.2, 35.6–48.9)	42.2	283 (63.5, 58.8–67.9)	36.5	< 0.0001
Yes	129 (57.8, 50.1–64.4)	100	163 (36.5, 32.1–41.2)	100	< 0.0001
Drug stores	73 (56.6, 47.6–65.3)	56.6	84 (51.5, 33.9–47.1)	51.5	0.4579
Village health posts	23 (17.8, 11.7–25.5)	74.4	55 (33.7, 22.4–34.6)	85.3	0.0035
Private clinics	15 (11.6, 6.7–18.5)	86.1	9 (5.5, 11.2–21.2)	90.8	0.0945
Public hospitals	16 (12.4, 7.3–19.4)	98.5	12 (7.4, 11.2–21.2)	98.2	0.2103
Others	2 (1.6, 0.2–5.5)	100	3 (1.8, 33.9–47.1)	100	0.8414

For malaria cases and non-malaria fever patients, N = 223 and N = 446, respectively, unless otherwise indicated

Percentages of the malaria patients who self-medicated first were significantly different between the subsamples enrolled in China (Table 2) and in Myanmar (Table 3) (P < 0.0001). Before attending a health facility with laboratory testing for malaria, 65.8% (100/152) of malaria cases recruited in China (Table 2) versus 43.7% (31/71) of those in Myanmar (Table 3) initially sought medication in sites without laboratory-based diagnosis (P = 0.0022). In terms of promptly seeking treatment for malaria, the percentages of malaria cases who sought treatment within 24 h were similar between in China (Table 2) and in Myanmar (Table 3) (P = 1.0000). The accumulated proportions of those who sought treatment in health facilities with laboratory testing for malaria within 48 h were significantly different between in China (Table 2) and in Myanmar (Table 3) (P < 0.0001). In China, 84.8% of malaria cases sought treatment in health facilities with laboratory testing for malaria within a week, with the longest time of 31 days after symptom onset (Table 2). In Myanmar, 98.6% (70/71) of malaria cases sought treatment in health facilities with laboratory testing for malaria within a week, with the longest time of 14 days after symptom onset (Table 3).

**Table 2:** Treatment-seeking behaviours between malaria cases and non-malaria fever patients recruited in China

Treatment-seeking behaviours	Malaria cases (%, 95%CI) N = 152	% accumulated	Non-malaria febrile patients (%, 95%CI) N = 304	% accumulated	*P value*
Time prior to attending health facilities with laboratory testing (hours)					
≤ 24(1 day)	22 (14.5, 9.3–21.1)	14.5	153 (50.3, 44.7.0–55.9)	50.3	< 0.0001
25–48(2 days)	23 (15.1, 8.8–20.3)	29.6	71 (15.4, 19.0–28.4)	73.7	0.0185
49–72(3 days)	23 (15.1, 9.8–21.8)	44.7	56 (18.4, 14.5–23.2)	92.1	0.4321
73–168(7 days)	61 (40.1, 32.3–48.4)	84.8	24 (7.9, 5.4–11.5)	100	< 0.0001
169–360 (15 days)	20 (13.2, 8.2–19.6)	98.0	0	100	–
> 360 (15 days)	3 (2.0, 1.1–7.5)	100	0	100	–
Self-medication prior to attending health facilities with laboratory testing					
Yes	62 (40.8, 23.9–49.1)	40.8	75 (24.7, 20.2–29.8)	24.7	0.0005
No	90 (59.2, 51.0–67.1)	100	229 (75.3, 70.2–79.8)	100	0.0005
Visited sites without laboratory testing for malaria prior to seeking appropriate treatment					
No visiting	52 (34.2, 25.2–45.8)	34.2	212 (69.7, 64.4–74.6)	69.7	< 0.0001
Yes	100 (65.8, 57.7–73.3)	100	92 (30.3, 25.4–35.6)	100	< 0.0001
Drug stores	51 (51.0, 41.7–62.2)	51.0	39 (42.4, 33.3–54.8)	42.4	0.3056
Village health posts	22 (22.0, 14.6–32.0)	73.0	39 (42.4, 33.3–54.8)	84.8	0.0029
Private clinics	14 (14.0, 8.0–22.8)	87.0	8 (8.7, 4.0–17.0)	93.5	0.3638
Public hospitals	11 (11.0, 5.7–19.2)	98.0	3 (3.3, 0.7–9.5)	96.7	0.0522
Others	2 (2.0, 0.1–6.0)	100	3 (3.3, 0.7–9.5)	100	0.6719

For malaria cases and non-malaria fever patients, N = 152 and N = 304, respectively, unless otherwise indicated

**Table 3:** Treatment-seeking behaviours between malaria cases and non-malaria fever patients recruited in Myanmar

Treatment-seeking behaviours	Malaria cases (%, 95%CI) N = 71	% accumulated	Non-malaria febrile patients (%, 95%CI) N = 142	% accumulated	*P value*
Time prior to attending health facilities with laboratory testing (hours)					
≤ 24(1 day)	10 (14.1, 7.0–24.4)	14.1	13 (9.2, 5.0–15.2)	9.2	0.3488
25–48(2 days)	32 (45.1, 33.2–57.3)	59.2	41 (28.9, 21.6–37.1)	38.0	0.0220
49–72(3 days)	15 (21.1, 9.8–21.8)	80.3	48 (33.8, 26.1–42.2)	71.8	0.0583
73–168(7 days)	13 (18.3, 10.1–29.2)	98.6	35 (24.7, 17.8–32.6)	96.5	0.3846
169–360 (15 days)	1 (1.4, 0.04–7.6)	100	4 (2.8, 0.8–7.1)	99.3	0.6669
> 360 (15 days)	0	100	1 (0.7, 0.02–3.9)	100	1.0000
Self-medication prior to attending health facilities with laboratory testing					
Yes	5 (7.0, 2.3–15.7)	7.0	8 (5.6, 2.5–10.8)	5.6	0.7636
No	66 (93.0, 84.3–97.7)	100	143 (94.4, 89.2–97.5)	100	0.7636
Visited sites without laboratory testing for malaria prior to seeking appropriate treatment					
No	40 (56.3, 44.1–68.1)	56.3	69 (48.6, 40.1–57.1)	48.6	0.3111
Yes	31 (43.7, 31.9–56.0)	100	73 (51.4, 43.9–59.9)	100	0.3111
Drug stores	22 (71.0, 42.7–85.8)	71.0	45 (61.6, 49.5–72.8)	61.6	0.5022
Village health posts	1 (3.2, 0.1–16.7)	74.2	16 (21.9, 13.1–33.1)	83.6	0.0197
Private clinics	1 (3.2, 0.1–16.7)	77.4	1 (1.4, 0.03–7.4)	84.9	0.5093
Public hospitals	5(16.1, 5.5–33.7)	93.6	9(12.3, 5.8–22.1)	97.3	0.7541
Others	2 (6.5, 0.8–21.42)	100	2 (2.7, 0.3–9.6)	100	0.5807

For malaria cases and non-malaria fever patients, N = 71 and N = 142, respectively, unless otherwise indicated

Percentages of the NMF patients with self-medication at first were significantly different between the subsamples enrolled in China (Table 2) and in Myanmar (Table 3) (P < 0.0001). Before attending a health facility with laboratory malaria testing, 75.3% of the NMF patients in China (Table 2) versus 51.4% (73/142) in Myanmar (Table 3) first sought medication from sites without laboratory-based diagnosis (P < 0.0001). In terms of promptly seeking treatment, proportions of the NMF patients who sought treatment within 24 h were significantly different between in China (Table 2) and in Myanmar (Table 3) (P < 0.0001). The accumulated proportion of the NMF patients seeking treatment in health facilities with laboratory malaria testing within 48 h was 73.7% in China (Table 2) compared to 38.0% in Myanmar (Table 3) (P < 0.0001). All 304 NMF patients recruited in China sought treatment in health facilities with laboratory malaria testing within a week, with the longest time being 7 days after symptom onset (Table 2). In Myanmar, 96.5% of the NMF patients sought treatment in health facilities with laboratory malaria testing within a week (P = 0.0435 compared to China), with the longest time being 30 days after symptom onset (Table 3).

### Influence factors of appropriate TSB

The multivariate LRA identified two independent risk factors associated with appropriate TSB in the malaria group. Malaria patients with Chinese nationality were more likely to delay in seeking appropriate treatment compared to those with other nationalities. Malaria patients living in urban areas were more likely to seek appropriate treatment compared to those living in rural areas (Table 4). Instead, the NMF patients with Chinese nationality were more likely to seek appropriate treatment than those with Burmese. The NMF patients from families with FWI > 3 were more likely to seek appropriate treatment too (Table 5).

**Table 4:** Factors associated with appropriate treatment-seeking behaviours (TSB) in the malaria patients*

Factors	No. appropriate TSB (%,95%CI)	Univariate OR (95%CI)	*P value*	Adjusted OR (95%CI)	*P value*
Nationalities					
Chinese (n = 130)	38 (29.2, 21.6–37.8)	0.40 (0.23–0.70)	0.0014	0.21 (0.07–0.68)	**0.0097**
Others (n = 93)￥	47 (50.5, 40.0–61.1)	1		1	
Sex					
Male (n = 163)	59 (36.2, 28.8–44.1)	0.74 (0.41–1.35)	0.3312	1.17 (0.53–2.57)	0.6909
Female (n = 60)	26 (43.3, 30.6–56.8)	1		1	
Age (years)					
≤ 30 (n = 121)	46 (38.0, 29.3–47.3)	0.99 (0.58–1.70)	0.97	0.47 (0.20–1.11)	0.0861
> 30(n = 102)	39 (38.2, 28.8–48.4)	1		1	
School education years					
> 6(n = 140)	47 (33.8, 25.8–42.0)	0.60 (0.34–1.04)	0.07	0.64 (0.31–1.33)	0.2296
≤ 6(n = 83)	38 (45.8, 34.8–57.1)	1		1	
Family wealth index					
> 3(n = 98)	30 (30.6, 21.7–40.7)	0.56 (0.32–0.98)	0.04	0.70 (0.33–1.45)	0.3342
≤ 3(n = 125)	55 (44.0, 35.1–53.2)	1		1	
Country staying ≤ 1 month					
China (n = 25)	8 (32.0, 14.9–53.5)	0.74 (0.30–1.80)	0.5051	0.83 (0.28–2.48)	0.7406
Myanmar (n = 198)	77 (38.9, 32.1–46.1)	1		1	
Location staying while the sick					
Urban (n = 83)	39 (47.0, 35.9–58.3)	1.81 (1.04–3.16)	0.0366	2.16 (1.06–4.39)	0.0337
Rural (n = 140)	46 (32.9, 25.2–41.3)	1		1	
Major cash sources					
Stable salary (n = 35)	16 (45.7, 28.8–63.4)	1.45 (0.70–3.00)	0.3189	0.64 (0.26–1.59)	0.3326
Others (n = 185)	68 (36.8, 29.8–44.1)	1		1	
Knowledge of malaria symptoms‡					
Yes (n = 178)	67 (37.6, 30.5–45.2)	0.91 (0.46–1.77)	0.7710	1.06 (0.41–2.70)	0.9107
No (n = 45)	18 (40.0, 25.7–55.7)	1		1	
Knowledge of malaria transmission§					
Yes (n = 150)	56 (37.3, 29.6–45.6)	0.90 (0.51–1.60)	0.7300	1.31 (0.57–2.98)	0.5237
No (n = 73)	29(39.7, 28.5–51.9)	1		1	
Knowledge of malaria prevention¶					
Yes (n = 161)	58 (36.0, 28.6–44.0)	0.73 (0.40–1.33)	0.3008	0.56 (0.23–1.38)	0.2050
No (n = 62)	27 (43.6, 31.0–56.7)	1		1	
Knowledge of nearby malaria endemic areas#					
Yes (n = 142)	54 (38.0, 30.0–46.5)	0.99(0.56–1.74)	0.9713	1.11 (0.47–1.47)	0.8109
No (n = 81)	31 (38.3, 27.7–49.7)	1		1	
Awareness of malaria prevention prior to entering nearby endemic areas					
Yes (n = 58)	25 (43.1, 30.2–56.8)	1.33 (0.72–2.44)	0.3640	1.00 (0.47–2.11)	0.9978
No (n = 165)	60 (36.4, 29.0–44.2)	1		1	
Family decision					
Wife or co- decision(n = 113)	44 (38.9, 29.9–48.6)	0.70(0.39–1.27)	0.24	1.25 (0.54–2.91)	0.5975
Others (n = 69)	33 (47.8, 35.6–59.9)	1		1	

^*^N = 223, unless otherwise indicated. ￥93 other nationalities included 92 Burmese and one Vietnamese; ‡ That a respondents knew fever as one of malaria symptoms was classified into “Yes”, otherwise “No”; § That a respondents knew mosquitoes transmitting malaria was classified into “Yes”, otherwise “No”; ¶That a respondents knew anyone of vector control and /or chemoprophylaxis was classified into “ Yes”, otherwise “No”; #That a respondents mentioned one of malaria endemic areas along China-Myanmar border was classified into “ Yes”, otherwise “No”

**Table 5:** Factors associated with appropriate treatment-seeking behaviours (TSB) in the non-malaria fever patients*

Factors	No. appropriate TSB (%,95%CI)	Univariate OR (95%CI)	*P value*	Adjusted OR (95%CI)	*P value*
Nationalities					
Chinese (n = 291)	220 (75.6, 70.3–80.4)	5.26 (3.44–8.04)	< 0.0001	3.49 (1.68–7.28)	0.0004
Burmese (n = 151)	56 (37.1, 29.4–45.3)	1		1	
Sex					
Male (n = 324)	204 (63.0, 57.6–68.0)	1.09 (0.70–1.67)	0.7090	0.90 (0.54–1.49)	0.6860
Female (n = 118)	72 (61.0, 51.6–69.9)	1		1	
Age (years)					
≤ 30 (n = 258)	153 (59.3, 53.0–65.4)	0.72 (0.48–1.07)	0.1069	1.38 (0.84–2.26)	0.2047
> 30(n = 184)	123 (66.9, 59.5–73.6)	1		1	
School education years					
> 6(n = 280)	184 (65.7, 59.8–71.3)	1.46 (0.98–2.17)	0.0624	0.69 (0.41–1.34)	0.1420
≤ 6(n = 162)	92 (56.8, 48.8–64.5)	1		1	
Family wealth index					
> 3(n = 243)	183 (75.3, 69.3- 80.6)	3.48 (2.32–5.20)	< 0.0001	1.71 (1.02–2.86)	0.0414
≤ 3(n = 199)	93 (46.7, 39.7- 53.9)	1		1	
Country staying ≤ 1 month					
China (n = 257)	203 (79.0, 73.5- 83.8)	5.77(3.79–8.78)	< 0.0001	1.40 (0.55–3.57)	0.4867
Myanmar (n = 185)	73 (39.5, 32.4- 46.9)	1		1	
Location staying while the sick					
Urban (n = 136)	91 (66.9, 58.3- 74.7)	1.32 (0.87–2.02)	0.1965	1.36 (0.79–2.35)	0.2674
Rural (n = 306)	185 (60.5, 54.9- 65.8)	1		1	
Major cash sources					
Stable salary (n = 14)	6 (42.9, 28.9- 82.3)	1.10 (0.62–1.95)	0.7380	1.02 (0.51–2.07)	0.9474
Others (n = 385)	238 (62.1, 57.2–66.9)	1		1	
Knowledge of malaria symptoms‡					
Yes (n = 306)	187 (61.1, 55.5–66.4)	0.82 (0.54–1.26)	0.3635	0.70 (0.43–1.13)	0.1437
No (n = 134)	88 (65.7, 57.0–73.7)	1		1	
Knowledge of malaria transmission§					
Yes (n = 334)	221 (66.2, 60.9–71.0)	1.02 (0.49–2.10)	0.9650	1.00 (0.48–2.06)	0.9980
No (n = 101)	50 (49.5, 39.4–59.6)	1		1	
Knowledge of malaria prevention¶					
Yes (n = 339)	213 (62.8, 57.6–67.8)	1.07 (0.68–1.69)	0.7600	1.03 (0.61–1.72)	0.9212
No (n = 103)	63 (61.2, 51.1–70.6)	1		1	
Knowledge of nearby malaria endemic areas#					
Yes (n = 234)	165 (70.5, 64.2–76.3)	2.09(1.41–3.09)	0.0002	1.15 (0.68–1.95)	0.5994
No (n = 208)	111 (53.4, 46.3–60.3)	1		1	
Awareness of malaria prevention prior to entering nearby endemic areas					
Yes (n = 141)	91 (64.5, 56.1–72.4)	1.15 (0.76–1.75)	0.5008	1.34 (0.80–2.25)	0.2650
No (n = 299)	183 (61.2, 55.4–66.8)	1		1	
Family decision					
Wife or co-decision(n = 279)	188 (63.4, 61.5–72.9)	1.76(1.18–2.62)	0.0052	1.21 (0.59–2.49)	0.5980
Others (n = 161)	87 (54.0, 46.0–61.9)	1		1	

^*^N = 446, unless otherwise indicated. ‡ That a respondents knew fever as one of malaria symptoms was classified into “Yes”, otherwise “No”; § That a respondents knew mosquitoes transmitting malaria was classified into “Yes”, otherwise “No”; ¶That a respondents knew anyone of vector control and /or chemoprophylaxis was classified into “ Yes”, otherwise “No”; #That a respondents mentioned one of malaria endemic areas along China-Myanmar border was classified into “ Yes”, otherwise “No”

## Discussion

### Malaria treatment-seeking behaviours

This study compared the TSB of the malaria cases to that of the NMF patients. The rate of malaria TSB at government facilities was the lowest (27.6%) in the WHO Southeast Asia Region (SEARO) in the world. However, the rate of accessing any sources of malaria treatment in SEARO communities was the highest (78.8%) out of all countries reported in 2016 [13]. The results of this study document a similar situation, showing inappropriate malaria TSB along the China-Myanmar border. Prior to attending a health facility with laboratory testing for malaria, more than half of malaria case patients sought treatment in drug stores, village health posts, and private clinics; and a high proportion of the malaria patients initially used self-medication, and delayed in appropriate TSB. This study was carried out from May 2016 to October 2017. Based on the results of this study, the malaria elimination program in China effectively communicated across communities, public health facilities, public hospitals, private medical sectors, and drug stores. The improvement of malaria treatment-seeking behaviours contributed to malaria-free certification in China [6, 16]. However, knowledge, awareness, and vigilance about malaria in communities and among health staff may fade after the malaria elimination. As existing malaria endemicity in the border areas of neighbouring countries, the Yunnan border area is still at a high risk of reestablishment of malaria transmission [9, 10]. Therefore, it is still necessary to promote appropriate TSB further in the area.

### Causes of inappropriate malaria TSB

Results of this study show that malaria TSB was inadequate in terms of punctuality and health facilities. The reasons accounted for the factors associated with delayed appropriate TSB are**:** (1) Malaria, as an infectious disease of poverty, frequently occurs in the underdeveloped world, and is also one of the causes that millions of households have failed to emerge out of poverty as they struggle with catastrophic health expenditures [2]. Some disadvantaged people in China still need to go to malaria endemic areas of neighbouring countries to make money, and then become infected with malaria parasites. Results of this study documented that households of malaria patients were poorer, had less schooling, and were more marginalized than households of the NMF patients. (2) Most parts of the Myanmar border areas neighbouring with China are managed by the local ethnic minority governments. The national health system of the Myanmar Union is unable to effectively cover these border areas, and the local ethnic health organizations cannot provide effective public health services to control infectious diseases [6, 9, 10, 12, 13, 21, 22]. In Myanmar, access to the retail sector was significantly higher than access to the public sector [24]. Chinese workers usually use self-medication due to the lack of health facilities for malaria treatment while staying locations in Myanmar. When self-medication does not work and then the disease becomes more severe, they have to return to China for diagnosis and treatment. (3) In China, over-the-counter (OTC) drugs are highly accessible. An investigation on TSB for dengue fever reported that two-thirds of participants (63.2%) would like to buy drugs from drug stores for dengue treatment at first [25]. (4) Following effective control of malaria, *P. vivax* has become the main parasite species along the China-Myanmar border [6, 9, 11]. Of 223 malaria cases, 210 (94.2%) cases were *P. vivax* malaria, 11 (4.9%) were *P. falciparum*, and 2 (0.9%) were mixed infections of *P. vivax* and *P. falciparum* [8]. The more benign symptoms of *P. vivax* malaria may be one of the causes of delays in TSB.

A number of studies have shown that the distance to the nearest health facility is one of the determinants associated with appropriate malaria treatment and diagnosis in Myanmar, Laos, India, Equatorial Guinea, Tanzania, and Indonesia [12, 26–30]. Almost all health facilities with laboratory testing for malaria are located at the township or higher-level health facilities along the China-Myanmar border area. The accessibility of laboratory testing for malaria remains a challenge for residents in the remote communities. Malaria elimination requires universal surveillance and a rapid public health response to malaria infection. As one application of the data from this investigation, villages which are either more than 5 km away by walking or more than 30 min away by motor vehicle are considered to be remote villages in Yunnan, China. Health or malaria workers are trained to use rapid diagnostic tests (RDTs) to detect febrile patients for malaria in the remote villages. When positive cases are detected by RDTs, the health or malaria workers report it to the County Center for Disease Control and Prevention (CDC), and then the county CDC will rapidly follow up with a microscopic investigation [5]. In the other hand, residents in the remote communities are usually less exposed to malaria information and have less access to health promotion activities, which may be another reason for the lower proportion of malaria patients who promptly sought treatment for malaria in health facilities with laboratory testing in the rural areas.

### Preventing reintroduction of malaria and border collaboration

Appropriate TSB is essential for surveillance and response to infectious diseases. Laboratory testing for malaria is mainly available in the public health sector along the China-Myanmar border, and it is expected that suspected malaria patients will seek treatment first in the public health facilities. Delays in seeking treatment can lead to adverse effects for both individuals and the community. For individuals, the delay increases the possibility of complications from malaria. At the community level, the delay increases the chance of onward transmission of the disease [31]. *Plasmodium vivax* is currently the main species of malaria parasites along the China-Myanmar border. Detection of *P. vivax* is not easy because parasitaemia can be much lower than that of *P. falciparum*, and a diagnostic test for liver-stage parasites of *P. vivax* is not currently available [11]. If malaria patients delay in appropriate TSB, the delayed detection can increase malaria onward transmission and exportation to neighboring countries. The increased importation of malaria might lead to a higher risk of malaria reintroduction in China [6]. Among the 223 respondents of the malaria group, 178 (79.8%), 150 (67.3%), and 142 (63.7%) of them respectively knew that fever was one of the malaria symptoms, that mosquitoes transmit malaria, and at least one of the malaria endemic areas. Additionally, 71 (31.8%) and 148 (66.4%) of them respectively acknowledged the need for chemoprophylaxis and preventing mosquito bites in the neighbouring country. This finding documents the need to improve people’s awareness of promptly seeking laboratory-based malaria diagnosis and appropriate treatment. Human migrants and anopheline mosquitoes infected with *Plasmodium* spp. can cause cross-border transmission of malaria [7]. Cross-border migrants are the most vulnerable group when it comes to malaria infection. They should be provided with information on individual protection against malaria infection in the Myanmar endemic areas, and the locations of health facilities with laboratory testing for malaria. A solid collaboration of cross-border malaria control between China and Myanmar is continuously necessary for the improvement of malaria diagnosis and treatment [6]. To improve the prognosis of malaria patients and reduce the chance of onward transmission, campaigns of information, education, and communication on awareness, knowledge, and vigilance of the community regarding malaria, and appropriate TSB, should be carried out among local communities, and Chinese migrants in Myanmar, so that people, including Chinese migrants, can directly seek and obtain appropriate malaria diagnosis and treatment in Myanmar.

## Limitations

The study was unavoidably limited by four obvious weaknesses. (1) Due to the instability of the ongoing political situation in the Myanmar border area and the fact that most malaria cases (> 80%) detected in Yunnan (China) were from the Laiza and nearby area, this study only recruited participants from the Laiza City Hospital in Myanmar [8]. The investigation may not fully reveal the TSB in the Myanmar border area; however, all the malaria cases enrolled in 37 health facilities in China were imported from the entire Myanmar border area, which can make up for some of this limitation. (2) In this study, all the participants were only enrolled in the health facilities. In cases where some patients purchased drugs from drug stores and their self-medication worked, they might not seek diagnosis and treatment from the health facilities, resulting in their exclusion from the study and creating selection bias. However, with the reduction of malaria burden, anti-malarial drugs are becoming less available in drug stores due to the lack of potential profit, making them only available in the designated public health facilities in China. This reduced the chance of the selection bias for the malaria group. (3) The participants were asked to remember things in detail during the past month, recall bias is unable to be avoided in some respondents. (4) Some participants declined to answer certain questions that they deemed to be sensitive, which could cause response bias.

## Conclusions

In summary, malaria TSB is inappropriate along the China-Myanmar border. Chinese citizenship and rural residence are two independent factors associated with appropriate malaria TSB. To improve the prognosis of individual malaria patients, eliminate malaria in Myanmar by 2030 and maintain malaria-free status in China, health education campaigns are continuously necessary to improve appropriate TSB in the cross-border collaboration programme. In the context of resurgent malaria in Myanmar and reduced border crossing limitations in the post COVID-19 era, it is urgently necessary to promote TSB for malaria control in Myanmar and preventing reestablishment of malaria transmission in Yunnan, China.

### Supplementary Information

**Additional file 1: Table S1. **Principal components for construction of the family wealth index (FWI). **Table S2. **Characteristics of malaria cases and non-malaria fever patients along China-Myanmar border. **Table S3. **Characteristics of malaria cases and non-malaria fever patients enrolled in China. **Table S4. **Characteristics of malaria cases and non-malaria fever patients enrolled in Myanmar.

## Data Availability

All relevant data are included in the manuscript and its supporting information files. The datasets generated and/or analyzed during the study are secured at the Yunnan Institute of Parasitic Diseases (YIPD). The details in the datasets are available upon official request to the director of the YIPD.

## Abbreviations

TSB: Treatment-seeking behaviour
LRA: Logistic regression analysis
NMF: Non-malaria febrile
AOR: Adjusted odds ratio
WHO: World Health Organization
GMS: Greater Mekong Subregion
CISDCP: China Information System for Disease Control and Prevention
KR1: Kachin Special Region I
KR2: Kachin Special Region II
KAR: Kokang Autonomous Region
SR2: Shan Special Region II
SR4: Eastern Shan Special Region IV
FWI: Family wealth index
RDT: Rapid diagnostic test
SEARO: WHO Southeast Asia Region
OTC: Over-the-counter
CDC: Center for disease control and prevention
CI: Confidence interval
