# Supplementary material for: Treatment-seeking behaviours of malaria patients versus non-malaria febrile patients along China-Myanmar border
Source: Malar J. 2023 Oct 14;22:309. doi: 10.1186/s12936-023-04747-4 (PMC10576386; doi:10.1186/s12936-023-04747-4)
Supplement: Supplementary file 1 — Additional file 1: Table S1. Principal components for construction of the family wealth index (FWI). Table S2. Characteristics of malaria cases and non-malaria fever patients along China-Myanmar border. Table S3. Characteristics of malaria cases and non-malaria fever patients enrolled in China. Table S4. Characteristics of malaria cases and non-malaria fever patients enrolled in Myanmar. [file 12936_2023_4747_MOESM1_ESM.docx]

**Additional file**

**Table S1 Principal components for construction of the family wealth index (FWI)**

| FWI | Housing characteristics | Transportation tools | Family belongings |
| --- | --- | --- | --- |
| 1 Most poor | Bamboo walls and sheet iron roofs | None | None or chickens |
| 2 Mid low | Wood walls and sheet iron roofs | Bicycles | Pigs or goats |
| 3 Middle | Brick walls, wood girders and terracotta roofs | Motorcycles | Cattle or horses |
| 4 Mid high | Brick concrete walls and terracotta roofs | Tractors | TV sets or refrigerators |
| 5 Least poor | Steel and concrete | Cars | Shops or elephants |

**Table S2 Characteristics of malaria cases and non-malaria fever patients along China-Myanmar border**

| Variables | No. malaria-cases  N = 223 (%, 95%CI) | No. non-malaria fever patients  N=446 (%, 95%CI) | *P* value |
| --- | --- | --- | --- |
| Demographics |  |  |  |
| Male sex | 163 (73.1, 66.8-78.8) | 326(73.1, 68.7-77.1) | 0.9263 |
| Age (years) |  |  |  |
| <16 | 27(12.1, 8.1-17.1) | 58(13.0, 10.1-16.6) | 0.8374 |
| ≥16 | 196(87.9, 82.9-91.9) | 388(87.0, 83.4-89.9) | 0.8374 |
| School education years | N=222 |  |  |
| ≤6 | 83(37.4, 31.0-44.1) | 163(36.6, 32.1-41.2) | 0.9774 |
| >6 | 139(62.6, 55.9-69.0) | 183(63.5, 58.8-67.9) | 0.9774 |
| Family wealth index |  |  |  |
| 1 Most poor | 6(2.7, 1.0-5.8) | 22(4.9, 3.2-7.5) | 0.2459 |
| 2 Mid low | 29(13.0, 8.9-5.1) | 66(14.8, 11.7-18.5) | 0.6107 |
| 3 Middle | 90(40.4, 33.9-47.1) | 111(24.9, 21.0-29.2) | 0.0001 |
| 4 Mid high | 63(28.3, 22.4-34.6) | 185(41.5, 36.9-46.2) | 0.0011 |
| 5 Least poor | 35(15.7, 11.2-21.2) | 62(13.9, 10.9-17.5) | 0.6138 |
| Nationality |  |  |  |
| China | 130(58.3, 51.5-64.8) | 295(66.1, 61.5-70.5) | 0.0571 |
| Myanmar | 92(41.3, 34.7-48.0) | 151(33.9, 29.5-38.5) | 0.0734 |
| Other (Vietnam） | 1(0.4, 0.01-2.5) | 0(0, 0-0.8) | 0.7235 |
| Country overnight in one month prior to attendance of health facilities | | | |
| China | 25(11.2, 7.4-16.1) | 261(58.5, 53.8-63.1) | <0.0001 |
| Myanmar | 198(88.8, 83.9-92.6) | 185(41.5, 36.9-46.2) | <0.0001 |
| Location stayed in one month prior to attendance of health facilities | | | |
| Home | 36(16.1,11.6-21.6) | 365(81.8, 77.9-85.2) | <0.0001 |
| IDP camp | 30(13.5, 9.3-18.6) | 29(6.5, 4.5-9.3) | 0.0045 |
| Field shelter huts | 41(18.4, 13.5-24.1) | 4(0.9, 0.3-2.4) | <0.0001 |
| Others（hotels, employer’s houses, friend’s and relatives’ home, and army camps） | 116(52.0, 45.3-58.7) | 48(10.8, 8.1-14.1) | <0.0001 |
| Residence ecology in one month prior to attendance of health facilities | | |  |
| Town | 83(37.2, 30.9-43.9) | 139(31.2, 26.9-35.7) | 0.1387 |
| Rural lowland and foothill | 140(62.8, 56.1-69.1) | 170(38.2, 33.6-42.8) | <0.0001 |
| Rural mid hill | 0(0, 0-1.6) | 79(17.7, 14.4-21.7) | <0.0001 |
| Rural upper hill | 0(0, 0-1.6) | 58(13.0, 10.1-16.6) | <0.0001 |
| Altitude |  |  |  |
| <500m | 163(73.1, 66.8-78.8) | 144(32.3, 28.0-36.8) | <0.0001 |
| 500-1000m | 57(25.6, 20.0-31.8) | 207(46.4, 41.7-51.2) | <0.0001 |
| >1000m | 3(1.4, 0.3-3.9) | 95(21.3, 17.6-25.4) | <0.0001 |
| Water body ≤100m |  |  |  |
| Yes | 214(96.0, 92.5-98.1) | 298(66.8, 62.2-71.1) | <0.0001 |
| No | 9(4.0, 1.7-7.5) | 148(33.2, 28.9-37.8) | <0.0001 |
| Paddy field | 3(1.4, 0.3-3.9) | 83(18.6, 15.1-22.5) | <0.0001 |
| Streamlets | 196(87.9, 82.9-91.9) | 137(30.7, 26.5-35.2) | <0.0001 |
| Water pools | 10(4.5, 2.2-8.1) | 58(13.0, 10.0-16.5) | 0.0010 |
| Water wells | 6(2.7, 1.0-5.8) | 20(4.5, 2.8-6.8) | 0.3579 |
| Residence proximity to vegetation in one month prior to attendance of health facilities | | | |
| Crops | 33(14.9, 10.5-20.2) | 130(29.2, 25.0-33.6) | 0.0001 |
| Shrub grass | 137(61.7, 55.0-68.1) | 122(27.4, 23.3-31.7) | <0.0001 |
| Forest | 34(15.3, 10.9-20.7) | 112(25.1, 21.2-29.4) | 0.0049 |
| Others | 18(8.1, 4.9-12.5) | 69(15.5, 12.2-19.2) | 0.0105 |
| Housing structure overnight in one month prior to attendance of health facilities | | | |
| Armoured concrete | 38(17.0, 12.4-22.6) | 126(28.3, 24.2-32.7) | 0.0021 |
| Brick and tile | 30(13.5, 9.3-18.6) | 171(38.3, 33.8-43.1) | <0.0001 |
| Wood and earth | 56(26.5, 20.8-32.8) | 55(12.3, 9.5-13.8) | <0.0001 |
| Wood and grass | 35(15.7, 11.2-21.2) | 89(20.0, 16.3-24.0) | 0.2182 |
| Thatched nuts | 61(27.4, 21.6-33.7) | 5(1.1, 0.4-2.8) | <0.0001 |
| Screened windows and doors | n=205 | n=401 |  |
| Yes | 17(8.3, 4.9-13.0) | 111(27.7, 23.4-32.3) | <0.0001 |
| No | 188(91.7, 87.1-95.1) | 290(72.3, 67.6-76.6) | <0.0001 |
| Measures against mosquito bites in home in one month prior to attendance of health facilities | | | |
| None | 45(20.2, 15.1-26.1) | 102(22.9, 19.1-27.1) | 0.4881 |
| Bed net | 169(75.8, 69.6-81.3) | 281(63.0, 58.3-67.5) | 0.0012 |
| Mosquito coil | 20(9.0, 5.6-13.5) | 94(21.1, 17.4-25.2) | 0.0001 |
| Spraying with insecticides | 0(0, 0-0.8) | 8(1.8, 0.8-3.5) | 0.0573 |
| Repellent | 9(4.0, 1.9-7.5) | 3(0.7, 0.2-2.1) | 0.0036 |
| Type of bed nets used | n=169 | n=281 |  |
| Untreated nets | 84(49.7, 41.9-57.5) | 232(82.6, 77.6-86.8) | <0.0001 |
| ITNs or LLINs | 85(50.3, 42.5-58.1) | 49(17.4, 13.2-22.4) | <0.0001 |
| Major household cash sources |  |  |  |
| Farming | 47(21.1, 15.9-27.0) | 207(46.4, 41.7-51.2) | <0.0001 |
| Working as day laborers | 12(5.4, 2.8-9.2) | 87(19.5, 16.0-23.6) | <0.0001 |
| Stable salary | 87(39.0, 32.6-45.8) | 61(13.7, 10.7-17.3) | <0.0001 |
| Trading and other | 57(25.6, 20.0-31.8) | 91(20.4, 16.8-24.4) | 0.1568 |
| Activities in one month prior to attendance of health facilities | | | |
| Trading | 36(16.1, 11.6-21.6) | 73(16.4, 13.1-20.2) | 0.9705 |
| Farming | 57(25.6, 20.0-31.8) | 216(48.4, 43.7-53.2) | <0.0001 |
| Building houses | 17(7.6, 4.5-11.9) | 19(4.3, 2.7-6.7) | 0.1019 |
| Building roads and mining | 11(4.9, 2.5-8.7) | 11(2.5, 1.3-4.5) | 0.1581 |
| Sight-seeing | 1(0.5, 0.01-2.5) | 5(1.1, 0.4-2.8) | 0.8721 |
| Visiting relatives and friends | 18(8.1, 4.9-12.5) | 5(1.1, 0.4-2.8) | <0.0001 |
| Lumbering | 2(0.9, 0.1-3.2) | 2(0.5, 0.1-1.8) | 0.6050 |
| Others | 81(36.3, 30.0-43.0) | 115(25.8, 21.8-30.2) | 0.0063 |
| Clinical and transmitting knowledge | |  |  |
| Clinical symptoms | 178(79.8, 73.9-84.9) | 312(70.0, 65.5-74.2) | 0.0354 |
| Transmitting | 150(67.3, 60.7-73.4) | 346(77.6, 73.4-81.4) | 0.0055 |
| Nearby endemic areas | 142(63.7, 57.0-70.0) | 237(53.1, 48.4-57.8) | 0.0121 |
| Preventive knowledge |  |  |  |
| Chemoprophylaxis | 71(31.8, 25.8-38.4) | 138(30.9, 26.7-35.5) | 0.8828 |
| Preventing mosquito bites | 148(66.4, 59.8-72.5) | 290(65.0, 60.4-69.5) | 0.7958 |
| Others | 9(4.0, 1.8-7.5) | 17(3.8, 2.2-6.0) | 0.9436 |
| Don’t know | 60(26.9, 21.2-33.2) | 105(23.5, 19.7-27.8) | 0.3919 |
| No consideration of prevention prior to entering endemic areas | 165(74.0, 67.7-79.6) | 301(67.7, 63.3-72.0) | 0.1521 |
| Family decision |  | n=444 |  |
| Refusing response | 41(18.4, 13.5-24.1) | 75(16.9, 14.0-20.8) | 0.7100 |
| Husband | 33(14.8, 10.4-20.2) | 43(9.7, 7.2-14.9) | 0.0670 |
| Wife | 12(5.4, 2.8-9.2) | 16(3.6, 2.1-5.9) | 0.3814 |
| Senior family member | 36(45.3, 20.5-43.1) | 44(9.9, 7.2-13.1) | 0.0270 |
| Co-decision | 101(45.3, 38.6-52.1) | 266(59.9, 55.2-64.5) | 0.1847 |
| Availability of foreigners nearby home | 73(32.9, 26.7-39.5) | 71(15.9, 12.7-19.7) | <0.0001 |

Note: For malaria cases and non-malaria fever patients, N = 223 and N = 446, respectively, unless otherwise indicated. IDP=internally displaced person; ITNs = insecticide-treated nets; LLINs = long-lasting insecticide nets.

**Table S3 Characteristics of malaria cases and non-malaria fever patients enrolled in China**

| Variables | No. malaria cases  N = 152 (%, 95%CI) | No. non-malaria fever patients  N=304 (%, 95%CI) | *P* value |
| --- | --- | --- | --- |
| Demographics |  |  |  |
| Male sex | 120 (79.0, 71.6- 85.1) | 240(79.0, 74.0-83.2) | 0.9154 |
| Age (years) |  |  |  |
| <16 | 10(6.6, 3.2- 11.8) | 22(7.2, 4.8-10.7) | 0.8244 |
| ≥16 | 142(93.4, 88.2- 96.8) | 282(92.8, 89.3-95.2) | 0.8244 |
| School education years |  |  |  |
| ≤6 | 59(38.8, 31.0- 47.1) | 82(27.0, 22.3-32.2) | 0.0132 |
| >6 | 93(61.2, 53.0-69.0) | 222(73.0, 67.8-77.7) | 0.0132 |
| Family wealth index |  |  |  |
| 1 Most poor | 0 | 1(0.3, 0.06-1.8) | 1.0000 |
| 2 Mid low | 3(2.0, 0.4-5.7) | 1(0.3, 0.06-1.8) | 0.1101 |
| 3 Middle | 69(45.4, 37.3- 53.7) | 77(25.3, 20.8-30.5) | 0.0001 |
| 4 Mid high | 52(34.2, 26.7- 42.3) | 169(55.6, 50.0-61.1) | 0.0011 |
| 5 Least poor | 28(18.4, 12.6- 25.5) | 56(18.4, 14.5-23.2) | 1.0000 |
| Overnight Country in one month prior to attendance of health facilities | | | |
| China | 21(13.8, 8.8- 20.3) | 259(85.2, 80.8-88.8) | <0.0001 |
| Myanmar | 131(86.2, 80.0- 91.2) | 45(14.8, 11.3-19.2) | <0.0001 |
| Location stayed in one month prior to attendance of health facilities | | | |
| Home | 8(5.3, 2.3-9.1) | 267(87.8, 83.7-91.0) | <0.0001 |
| IDP camp | 14(9.2, 5.1-15.0) | 3(1.0, 0.3-2.9) | <0.0001 |
| Field shelter huts | 39(25.7, 18.9- 33.4) | 1(0.3, 0.06-1.8) | <0.0001 |
| Others | 91 (60.0, 51.6- 67.7) | 33(10.9, 7.8-14.9) | <0.0001 |
| Features of staying site in one month prior to attendance of health facilities | | |  |
| Town | 46(30.3, 23.1-38.2) | 98(32.2, 27.2-37.7) | 0.7486 |
| Rural lowland and foothill | 106(69.7, 61.7-76.9) | 144(47.4, 41.3-53.0) | <0.0001 |
| Rural mid hill | 0(0, 0-1.8) | 39(12.8, 9.5-17.1) | <0.0001 |
| Rural upper hill | 0(0, 0-1.8) | 23(7.6, 5.1-11.1) | 0.0001 |
| Altitude |  |  |  |
| <500m | 110(72.4, 64.5-79.3) | 78(25.7, 21.1-30.9) | <0.0001 |
| 500-1000m | 42(27.6, 20.7-35.5) | 164(54.0, 48.3-59.5) | <0.0001 |
| >1000m | 0(0, 0-1.8) | 62(20.4, 16.3-25.3) | <0.0001 |
| Water body ≤100m |  |  |  |
| Yes | 143(94.1, 89.1-97.3) | 191(62.8, 57.3-68.1) | <0.0001 |
| No | 9(5.9, 2.7-10.9) | 113 (37.2, 31.9-42.7) | <0.0001 |
| Paddy field | 3(2.1, 0.4-6.0) | 81(42.4, 35.3-49.8) | <0.0001 |
| Streamlets | 138(95.8, 91.2-98.5) | 72(37.7, 30.8-45.0) | <0.0001 |
| Water pools | 3(2.1, 0.4-6.0) | 36(18.9, 13.6-25.1) | 0.0001 |
| Water wells | 0(0, 0-1.8) | 2(1.1, 0.1-3.7) | 0.5546 |
| Residence proximity to vegetation in one month prior to attendance of health facilities | | | |
| Crops | 29(19.1, 13.2-26.2) | 121(39.8, 34.5-45.4) | 0.0001 |
| Shrub grass | 83(54.6, 46.3-62.7) | 46(15.1, 11.5-19.6) | <0.0001 |
| Forest | 26(17.1, 11.5-24.1) | 59(19.4, 15.4-24.2) | 0.6108 |
| Others | 14(9.2, 5.1-15.0) | 78(25.7, 21.1-30.9) | <0.0001 |
| Housing structure overnight in one month prior to attendance of health facilities | | | |
| Armoured concrete | 15(9.9, 5.6-15.8) | 103(33.9, 28.8-39.4) | <0.0001 |
| Brick and tile | 16(10.5, 6.1-16.5) | 153 (50.3, 44.7-55.9) | <0.0001 |
| Wood and earth | 53(34.9, 27.3-43.0) | 42(12.3, 10.4-18.2) | <0.0001 |
| Wood and grass | 9(5.9, 2.7-10.9) | 2(0. 7, 0.2-2.4) | 0.0012 |
| Thatched cottages | 59(38.8, 31.0-47.1) | 4(1.3, 0.5-3.3) | <0.0001 |
| Screened windows and doors | n=147 | n=283 |  |
| Yes | 9(6.1, 2.8-11.3) | 103(36.4, 30.8-42.3) | <0.0001 |
| No | 138(93.9, 88.7-97.2) | 180(63.6, 57.7-69.2) | <0.0001 |
| Measures against mosquito bites in home in one month prior to attendance of health facilities | | | |
| None | 41(27.0, 20.1-34.8) | 79(26.0, 21.4-31.2) | 0.8224 |
| Bed net | 105(69.1, 61.1-76.3) | 147(48.4, 42.8-54.0) | <0.0001 |
| Mosquito coil | 6(4.0, 1.5-8.4) | 78 (25.7, 21.1-30.9) | <0.0001 |
| Type of bed nets used | n=105 | n=168 |  |
| Untreated nets | 47(44.7, 35.1-54.8) | 159(94.6, 90.1-97.5) | <0.0001 |
| ITNs or LLINs | 58(55.2, 45.2-65.0) | 9(5.4, 2.2-12.3) | <0.0001 |
| Major cash sources |  |  |  |
| Farming | 35(23.0, 16.6-30.5) | 169(55.6, 50.0-61.1) | <0.0001 |
| Working as day laborers | 75(49.3, 41.2-57.6) | 47(15.5, 11.8-20.0) | <0.0001 |
| Stable salary | 6(4.0, 1.5-8.4) | 33(10.9, 7.8-14.9) | 0.0125 |
| Trading and other | 36 (23.7, 17.2-31. 3) | 55(17.1, 13.6-23.1) | 0.1724 |
| Activities in one month prior to attendance of health facilities | | | |
| Trading | 33(21.7, 15.4-29.1) | 62(20.4, 16.3-25.3) | 0.8069 |
| Farming | 53(34.9, 27.3-43.0) | 165(54.3, 48.7-59.8) | 0.0001 |
| Building houses | 17 (11.2, 6.7-17.3) | 14(4.6, 2.8-6.6) | 0.0163 |
| Building roads and mining | 9(5.9, 2.7-10.9) | 12(3.9, 1.8-8.3) | 0.7999 |
| Sight-seeing | 1(0.7, 0.02-3.6) | 5(1.6, 0.7-3.8) | 0.6686 |
| Visiting relatives and friends | 18 (11.8, 7.2-18.1) | 5(1.6, 0.7-3.8) | <0.0001 |
| Lumbering | 2(1.3, 0.2-4.7) | 2(0.7, 0.2-2.8) | 0.6028 |
| Others | 19(12.5, 7.7-18.8) | 44(14.5, 11.8-18.9) | 0.6662 |
| Clinical and malaria transmission knowledge | |  |  |
| Clinical symptoms | 118(77.6, 70.2-84.0) | 207(68.1, 62.7-73.1) | 0.0371 |
| Malaria transmission | 105(69.1, 61.1-76.3) | 233(76.6, 71.6-81.1) | 0.0895 |
| Nearby endemic areas | 111(73.0, 65.2-79.9) | 201(66.1, 60.6-71.2) | 0.1646 |
| Preventive knowledge |  |  |  |
| Chemoprophylaxis | 69(45.4, 37.3-53.7) | 130(42.8, 37.3-48.4) | 0.6173 |
| Preventing mosquito bites | 41(27.0, 20.1-34.8) | 100(32.9, 27.9-38.4) | 0.2370 |
| Don’t know | 42(27.6, 20.7-35.5) | 74(24.3, 19.9-29.5) | 0.1443 |
| No consideration of prevention prior to entering endemic areas | 130(85.5, 78.9-90.7) | 209(69.2, 63.8-74.1) | 0.0001 |
| Family decision |  |  |  |
| Refusing response | 7(4.6, 1.4-8.8) | 70(23.0, 18.7-28.1) | <0.0001 |
| Husband | 18(11.8, 7.2-18.1) | 4(1.3, 0.5-3.3) | <0.0001 |
| Wife | 4(2.6, 0.6-6.6) | 3(1.0, 0.3-2.9) | 0.2288 |
| Senior family member | 2(1.3, 0.2-4.7) | 5(1.6, 0.7-3.8) | 1.0000 |
| Co-decision | 87 (57.2, 49.0-65.2) | 222(73.0, 67.8-77.7) | 0.0009 |
| Availability of foreigners nearby home | 51(33.8, 26.3-41.9) | 54(17.8, 13.9-22.5) | 0.0002 |

Note: For malaria cases and non-malaria fever patients, N = 152 and N = 304, respectively, unless otherwise indicated. IDP=internally displaced person; ITNs = insecticide-treated nets; LLINs = long-lasting insecticide nets.

**Table S4 Characteristics of malaria cases and non-malaria fever patients enrolled in Myanmar**

| Variables | No. malaria-cases  N = 71 (%, 95%CI) | No. non-malaria fever patients  N=142 (%, 95%CI) | *P* value |
| --- | --- | --- | --- |
| Demographics |  |  |  |
| Male sex | 43 (60.6, 48.3-72.7) | 86(60.6, 52.0-68.7) | 1.000 |
| Age (years) |  |  |  |
| <16 | 17(24.0, 14.6-35.5) | 36(25.4, 18.4-33.3) | 0.9553 |
| ≥16 | 54(76.1, 64.5-85.4) | 106(74.6, 66.7-81.6) | 0.9553 |
| School education years |  |  |  |
| ≤6 | 5(7.0, 2.3- 15.7) | 9(6.3, 2.9-11.7) | 0.7717 |
| >6 | 66(93.0, 84.3-97.7) | 133(93.7, 88.3-97.1) | 0.7717 |
| Family wealth index |  |  |  |
| 1 Most poor | 6(8. 5, 3.2-17.5) | 21(14.8, 9.4-21.7) | 0.2748 |
| 2 Mid low | 26(36.6, 25.5-48.9) | 65(45.8, 37.4-54.3) | 0.2600 |
| 3 Middle | 21(29.6, 19.3-41.6) | 34(23.9, 17.2-31.8) | 0.4718 |
| 4 Mid high | 11(15.5, 8.0-26.0) | 16(11.3, 6.6-17.7) | 0.5123 |
| 5 Least poor | 7(9.9, 4.1-19.3) | 6(4.2, 1.6-9.0) | 0.1883 |
| Nationality |  |  |  |
| China | 1(1.4, 0.04-7.6) | 4(2.8, 0.8-7.1) | 0.8729 |
| Myanmar | 70(98.6, 92.40-99.9) | 138(97.2, 92.9-99.2) | 0.8729 |
| Country overnight in one month prior to attendance of health facilities | | |  |
| China | 4(5.6, 1.6-13.8) | 2(1.4, 0.2-5.0) | 0.1942 |
| Myanmar | 67(94.4, 86.20-98.4) | 140(98.6, 95.0-99.8) | 0.1942 |
| Location stayed ≤ 1 month prior to malaria attack | |  |  |
| Home | 28(39.4, 28.0-51.8) | 98(69.0, 60.7-76.5) | 0.0001 |
| IDP camp and field shelters | 18(25.4, 15.8-37.1) | 29(20.4, 14.1-28.0) | 0.4410 |
| Others | 25(35.2, 24.2-47.5) | 15(10.6, 6.0-16.8) | <0.0001 |
| Residence ecology in one month prior to attendance of health facilities | | |  |
| Laiza city | 37(52.1, 39.9-64.1) | 41(28.9, 21.6-37.1) | 0.0056 |
| Rural area | 34(47.9, 35.9-60.1) | 101(71.1, 62.9-78.4) | 0.0056 |
| Altitude <500m | 53(74.6, 62.9-84.2) | 66(46.5, 38.1-55.0) | 0.0002 |
| Altitude≥500m | 18(25.4, 15.8-37.1) | 76(53.5, 45.0-61.9) | 0.0002 |
| Streamlets≤100m | 58(81.7, 70.7-89.9) | 65(60.2, 50.3-69.5) | <0.0001 |
| Other water source≤100m | 13(18.4, 10.1-29.3) | 43(30.3, 22.9-38.5) | 0.0880 |
| Residence proximity to vegetation in one month prior to attendance of health facilities | | |  |
| Crops | 7(9.9, 4.1-19.3) | 9(6.3, 2.9-11.7) | 0.5120 |
| Shrub grass and forest | 62(87.3, 77.3-94.0) | 129(90.8, 84.9-95.0) | 0.5774 |
| Others | 2(2.8, 0.3-9.8) | 4(2.8, 0.8-7.1) | 0.6620 |
| Housing structure overnight in one month prior to attendance of health facilities | | |  |
| Armoured concrete | 23(32.4, 21.8-44.6) | 23(16.2, 10.6-23.3) | 0.0114 |
| Brick and tile | 14(19.7, 11.2-30.9) | 18(12.7, 7.7-19.3) | 0.2491 |
| Wood, earth and Thatched cottages | 34(47.9, 35.9-60.1) | 101(71.1, 62.9-78.4) | 0.0015 |
| Screened windows and doors | n=58 | n=118 |  |
| Yes | 8(13.8, 6.2-25.4) | 8(6.8, 3.0-12.9) | 0.2322 |
| No | 50(86.2, 74.6-93.9) | 110(93.2, 87.1-97.0) | 0.3408 |
| Measures against mosquito bites in one month prior to attendance of health facilities | | |  |
| None | 4(5.6, 1.6-13.8) | 23(16.2, 10.6-23.3) | 0.0493 |
| Bed nets | 64(90.1, 80.7-95.9) | 113(79.6, 72.0-85.9) | 0.0809 |
| Mosquito coil | 11(15.5, 8.0-26.0) | 15(10.6, 6.0-16.8) | 0.4156 |
| Others | 1(1.4, 0.04-7.6) | 1(0.7, 0-3.9) | 0.8017 |
| Type of bed nets used | n=64 | n=113 |  |
| Untreated nets | 37(57.8, 44.8-70.1) | 73(64.6, 55.1-73.4) | 0.4633 |
| ITNs or LLINs | 27(42.2, 29.9-55.2) | 40(35.4, 26.6-45.0) | 0.4633 |
| Major household cash sources |  |  |  |
| Farming | 12(16.9, 9.1-27.7) | 39(27.5, 20.3-35.6) | 0.1253 |
| Working as day laborers | 12(16.9, 9.1-27.7) | 40(28.2, 21.0-36.3) | 0.1077 |
| Stable salary | 30(42.3, 30.6-54.6) | 28(19.7, 13.5-27.2) | 0.0009 |
| Trading and other | 21(29.8, 19.8-40.9) | 35(24.6, 17.8-32.6) | 0.5450 |
| Clinical and transmitting knowledge | |  |  |
| Clinical symptoms | 60(84.5, 74.0-92.0) | 105(73.9, 65.9-80.9) | 0.1175 |
| Transmitting | 45(63.4, 51.1-74.5) | 112(78.9, 71.2-85.3) | 0.0241 |
| Nearby endemic areas | 31(43.7, 31.9-56.0) | 36(25.4, 18.4-33.3) | 0.0106 |
| Preventive knowledge |  |  |  |
| Chemoprophylaxis | 2(2.8, 0.3-9.8) | 8(5.6, 2.5-10.8) | 0.5669 |
| Preventing mosquito bites | 51(71.8, 59.9-81.9) | 101(71.1, 62.9-78.4) | 0.9573 |
| Others | 9(12.7, 6.0-22.7) | 17(12.0, 7.1-18.5) | 0.9410 |
| Don’t know | 18(25.4, 15.8-37.1) | 30(21.1, 14.7-28.8) | 0.6019 |
| No consideration of prevention | 36(50.7, 38.6-62.8) | 50(35.2, 27.4-43.7) | 0.04294 |
| Family decision |  | n=135 |  |
| Husband | 15(21.1, 12.3-32.4) | 39(28.9, 21.4-37.3) | 0.2996 |
| Wife or co-decision | 22(31.0, 20.5-43.1) | 57(42.1, 33.8-51.0) | 0.1540 |
| Senior family member | 34(47.9, 35.9-60.1) | 39(28.9, 21.4-37.3) | 0.0106 |

Note: For malaria cases and non-malaria fever patients, N = 71 and N = 142, respectively, unless otherwise indicated. IDP=internally displaced person; ITNs = insecticide-treated nets; LLINs = long-lasting insecticide nets.
